# Overexpressed Histocompatibility Minor 13 was Associated with Liver Hepatocellular Carcinoma Progression and Prognosis

**DOI:** 10.1155/2022/7067743

**Published:** 2022-10-03

**Authors:** Rui-Qing Zong, Hong-Yan Zhang, Xiao-Ying Li, Yi-ran Li, Ying Chen

**Affiliations:** ^1^Department of Emergency, Ruijin Hospital, Shanghai Jiao Tong University School of Medicine, Shanghai, China; ^2^Department of Intensive Care Medicine, Eastern Hepatobiliary Surgery Hospital, The Third Affiliated Hospital of Naval Medical University, Shanghai, China

## Abstract

Among primary liver carcinoma cases, the proportion of liver hepatocellular carcinoma (LIHC) cases is 75%–85%. Current treatments for LIHC include chemotherapy, surgical excision, and liver transplantation, which are effective for early LIHC treatment. Nevertheless, the early symptoms of liver carcinoma are atypical, so a large proportion of LIHC patients are diagnosed at an advanced stage. Histocompatibility minor 13 (HM13), located in the endoplasmic reticulum, is responsible for catalysing the hydrolysis of some signal peptides after cleavage from the precursor protein. Here, we studied the role of HM13 in LIHC development through bioinformatics analysis. Database analysis showed that HM13 was of great significance for LIHC tumorigenesis. Compared to normal liver tissues, HM13 expression was increased to a greater extent in LIHC tissues. After analysis of Kaplan‒Meier plotter and Gene Expression Profiling Interactive Analysis (GEPIA) datasets, we discovered that highly expressed HM13 exhibited an association with shorter overall survival (OS), disease-free survival (DFS), and disease-specific survival (DSS). We conducted Gene Ontology (GO) and Kyoto Encyclopedia of Genes and Genomes (KEGG) analyses to analyse HM13-related genes, and the data indicated that these genes obviously participated in rRNA processing, ribosome biogenesis, spliceosome, Huntington's disease, and ATP-dependent helicase activity. The Cell Counting Kit-8 (CCK-8) assay and Transwell assay showed that reducing HM13 expression hindered LIHC cell proliferation, migration, and invasion. In conclusion, these findings indicate that HM13 is a biomarker and is related to the poor prognosis of LIHC. Our results are conducive to discovering new targets for LIHC treatment.

## 1. Background

It is predicted that liver carcinoma will be the sixth most common carcinoma once diagnosed and the fourth main inducer of carcinoma mortality worldwide [1, 2], with approximately 841,000 new cases and 782,000 deaths occurring annually [3]. Among primary liver carcinomas, the proportion of liver hepatocellular carcinoma (LIHC) cases is 75%–85% [4]. At present, curative treatments for LIHC comprise chemotherapy, surgical operation, and liver transplantation, which are efficacious at an early stage of LIHC [5–7]. Nevertheless, the early manifestation of liver carcinoma is uncommon, and most LIHC patients are diagnosed at an advanced stage [8]. Advanced-stage LIHC has become a disease that cannot be cured by surgery, and only a few patients can receive radical resection [9]. Moreover, due to the high frequency of metastasis and recurrence of LIHC, the prognosis of LIHC patients after treatment is poor. These challenges make it necessary to identify potential biomarkers and discover new targets to uncover more effective treatments for LIHC.

Presently, the pathogenesis of LIHC metastasis is not clear, which may be related to the unusual expression of multiple oncogenes and neoplasm inhibitor genes. Some molecular biomarkers have presented a relationship with LIHC progression based on the rapid development of molecular biotechnology. Seeking new molecular markers of encoding genes is conducive to understanding the etiology of LIHC and evaluating the prognostic status of early-stage LIHC.

Using bioinformatics analyses, we found that histocompatibility minor 13 (HM13) was associated with LIHC. HM13, located in the endoplasmic reticulum, catalyses the proteolysis of signal peptide cleavage from the precursor protein [10]. This capability of HM13 is essential for producing the immune system-recognised human lymphocyte antigen *E* epitope and processing the core protein of the hepatitis C virus. Recent studies have shown that HM13 may be related to the pathogenesis of some carcinomas. For instance, Goovaerts et al. found that HM13 expression was upregulated in breast carcinoma [11].

Nevertheless, the role of HM13 in LIHC remains unclear. Here, we employed data mining technology to identify HM13 as a new prognostic indicator in LIHC. The association between HM13 expression and LIHC was validated by Kaplan‒Meier (KM) plotter and Gene Expression Profiling Interactive Analysis (GEPIA) datasets. HM13-related gene enrichment was analysed by Gene Ontology (GO) terms and Kyoto Encyclopedia of Genes and Genomes (KEGG) pathways. Our results suggest that HM13 may increase the occurrence risk of LIHC and aggravate the prognostic status of patients.

## 2. Material and Methods

### 2.1. Data Sources

This study analysed two datasets. The first was GEPIA, and we obtained the clinical and survival information of LIHC patients from GEPIA (https://gepia.cancer-pku.cn/). The second was KM-plotter (https://kmplot.com/analysis/). We acquired HM13 gene expression profiles and Kaplan‒Meier survival curves of the overall survival (OS), disease-free survival (DFS), and disease-specific survival (DSS) of LIHC patients from the two databases.

### 2.2. Identification of the Signalling Pathways

We conducted GO enrichment and KEGG pathway analyses to discover the probable signalling pathways of HM13-associated genes in LIHC. We applied GEPIA to obtain 1000 HM13-associated genes in LIHC. Biological process (BP), cell component (CC), and molecular function (MF) categories, as well as KEGG pathway analysis, were conducted using the database for annotation, visualisation, and integrated discovery (DAVID, https://david.ncifcrf.gov/tools.jsp).

### 2.3. Cell Culture and Transfection

LO2, LM3, Hep3b, MHCC-97H, Huh7, and SMMC-7221 cells were acquired from the American Type Culture Collection (ATCC). LM3, SMMC-7221, and MHCC-97H cells were maintained in Roswell Park Memorial Institute 1640 (RPMI-1640, Gibco) medium with 10% foetal bovine serum (FBS, Sigma‒Aldrich) and 1% penicillin/streptomycin (Life Technology) at 37°C with 5% CO2. LO2, Huh7, and Hep3b cells were kept in Dulbecco's modified Eagle's medium (DMEM, Gibco) with 10% FBS (Sigma‒Aldrich) and 1% penicillin/streptomycin (Life Technology). Control siRNA (si-NC, GenePharma) or siRNAs specific for HM13 (si-HM13, GenePharma) were transfected into Huh7 and SMMC-7721 cells by Lipofectamine 2000 Reagent (Invitrogen) and Opti-MEM medium (Life Technology) at the indicated times.

### 2.4. Extraction and Quantitation of RNA

TRIzol (TAKARA, Japan) was applied to harvest overall RNA following the manufacturer's instructions. Harvested RNA was quantified by a NanoDrop ND-2000 spectrophotometer and then transcribed into cDNA utilising PrimeScript RT Master Mix (TAKARA, Japan). Real-time polymerase chain reaction (PCR) was conducted on a 7500 Fast Real-Time PCR system (Applied Biosystems, USA) with Synergy Brands (SYBR) Premix Ex Taq (TAKARA, Japan). The PCR parameters for RT‒PCR were 95°C for 2 min and 39 cycles of 95°C for 20 s, 58°C for 30 s, and 72°C for 30 s. Relative gene expression was calculated by the 2^−ΔΔCt^ method. Glyceraldehyde-3-phosphate dehydrogenase (GAPDH) was an internal reference.

### 2.5. Cell Counting Kit-8 (CCK-8) Assay

A total of 2 × 103 si-HM13-or si–NC–transfected Huh7 and SMMC-7721 cells were inoculated into each well of a 96-well plate. We detected cell viability per well after adding CCK-8 solution (Dojindo, Japan) at specific times. The absorbance at 450 nm was detected utilising a microplate reader (Tecan Group Ltd.).

### 2.6. Transwell Assay

For the migration assay, 10 × 104 cells of stable cell lines (Huh7 si-NC, Huh7 si-HM13, SMMC-7721 si-NC, and SMMC-7721 si-HM13) in 250 *μ*l of serum-free medium were inoculated in the upper chamber of 8-*μ*m Transwell inserts (BD Biosciences, USA). Then, 500 *μ*l of medium with 10% FBS was added to the lower chamber. We removed Huh7 si-HM13 and SMMC-7721 si-HM13 cells in the upper chamber with a cotton swab. PBS was applied to wash the migrated cells on the underside. The migrated cells were then fixed with methanol (Solarbio, China) for 10 min and stained with 10 *μ*g/mL diamidino-phenyl-indole (DAPI, Solarbio, China) for 10 min. Positive cells in 5 random fields were photographed under a 200× inverted microscope DMI4000 B (Leica, Germany) and counted. For the invasion assay, the transwell chamber was pretreated with Matrigel, and the other experimental procedures were the same.

### 2.7. Statistical Analyses

Statistical Product and Service Solutions (SPSS) 22.0 (Chicago, USA) and *R* version 3.6.0 were applied to analyse the data. The differences existing between two groups or among multiple groups were assessed by Student's *t*-test and analysis of variance (ANOVA), respectively. The KM method was employed to plot survival curves. The log-rank test was utilised to compare differences. A significant difference was indicated when *P* < 0.05.

## 3. Results

### 3.1. Analysis of the Differentially Expressed HM13 in Carcinoma and Normal Tissues

**Figure 1:**
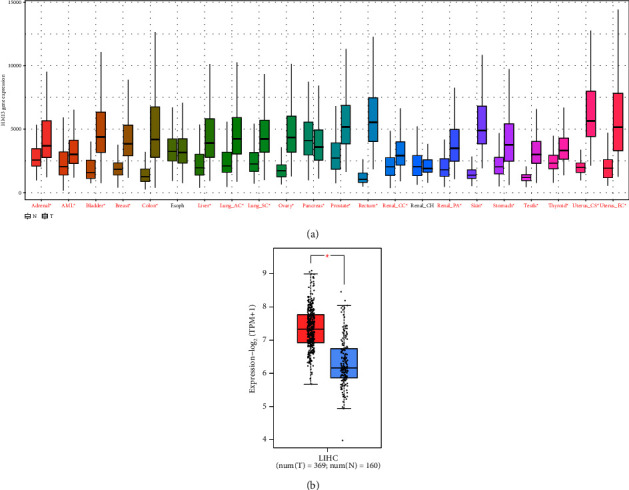
Comparison of HM13 expression in the tissues of carcinoma and normal. (a) Analysis of HM13 expression in different cancer. (b) Highly expressed HM13 was shown in LIHC tissues. (^*∗*^*P* < 0.05).

We used the Kaplan‒Meier database to analyse HM13 expression in different tissues. Our data showed that compared with normal tissues, HM13 expression was higher in adrenal, acute myeloid leukaemia (AML), bladder, breast, colon, liver, lung adenocarcinoma (Lung_AC), lung sarcomatoid carcinoma (Lung_SC), ovary, pancreas, prostate, rectum, renal clear cells (Renal_CC), renal congenital hydronephrosis (Renal_CH), renal pretubular aggregate (Renal_PA), skin, stomach, testis, thyroid, uterine caesarean section (Uterus_CS), and uterine endometrial cancer (Uterus_EC) (Figure 1(a)). The GEPIA database analysis indicated that HM13 expression was upregulated in LIHC compared with normal tissues (Figure 1(b)).

### 3.2. High HM13 Expression Was Significantly Associated with Poor Prognosis of LIHC

**Figure 2:**
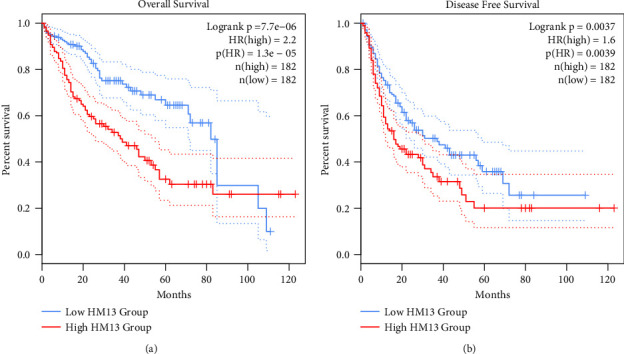
Higher expression of HM13 exhibited an association with worse OS and DFS. The survival curve showed that the (a) OS and (b) DFS of patients with highly expressed HM13 were lower compared to that of patients with lowly expressed HM13 in the GEPIA database.

We obtained the OS and DFS data of 364 LIHC patients in the GEPIA database. Patients with high HM13 expression displayed shorter OS than those with low HM13 expression (Figure 2(a)). In addition, shorter DFS was also observed in patients with high HM13 expression than in those with low HM13 expression (Figure 2(b)). Our findings showed that high expression of HM13 exhibited a close relationship with poor prognostic status of LIHC.

### 3.3. High Expression of HM13 was a Predictor of LIHC Prognosis

**Figure 3:**
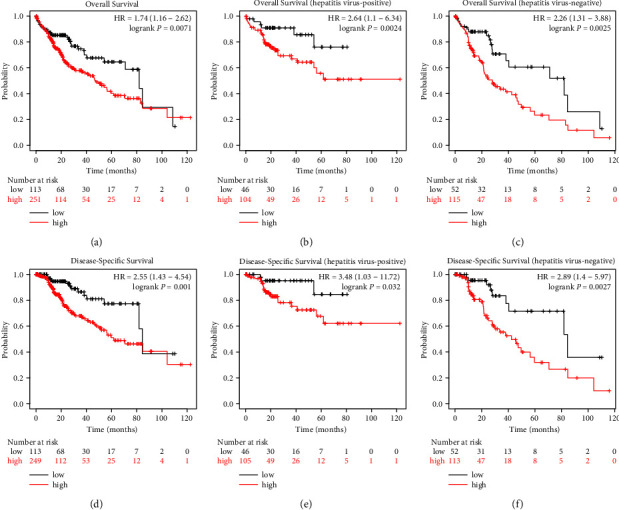
Kaplan–Meier survival curves of LIHC patients with highly expressed and lowly expressed HM13. (a) Survival curves of OS in LIHC patients in KM-plotter database. (b–c) Survival curves of OS in LIHC patients with or without hepatitis virus in KM-plotter database. (D) Survival curves of DSS in LIHC patients in KM-plotter database. (e–f) Survival curves of DSS in LIHC patients with or without hepatitis virus in KM-plotter database.

To further evaluate the prognostic value of HM13, including OS and DSS, in LIHC with different HM13 levels, we used the KM-plotter database. LIHC patients with high HM13 expression displayed shorter OS and DSS than those with low HM13 expression (Figures 3(a) and 3(d)). The OS and DSS of LIHC patients with hepatitis virus in the low HM13 group were significantly higher than those in the high HM13 group (Figures 3(b) and 3(e)). We observed a similar result in LIHC patients without hepatitis virus (Figures 3(c) and 3(f)). These results suggest that high HM13 expression is a predictor of poor LIHC prognosis.

### 3.4. Identification of the Signalling Pathways Involved

**Figure 4:**
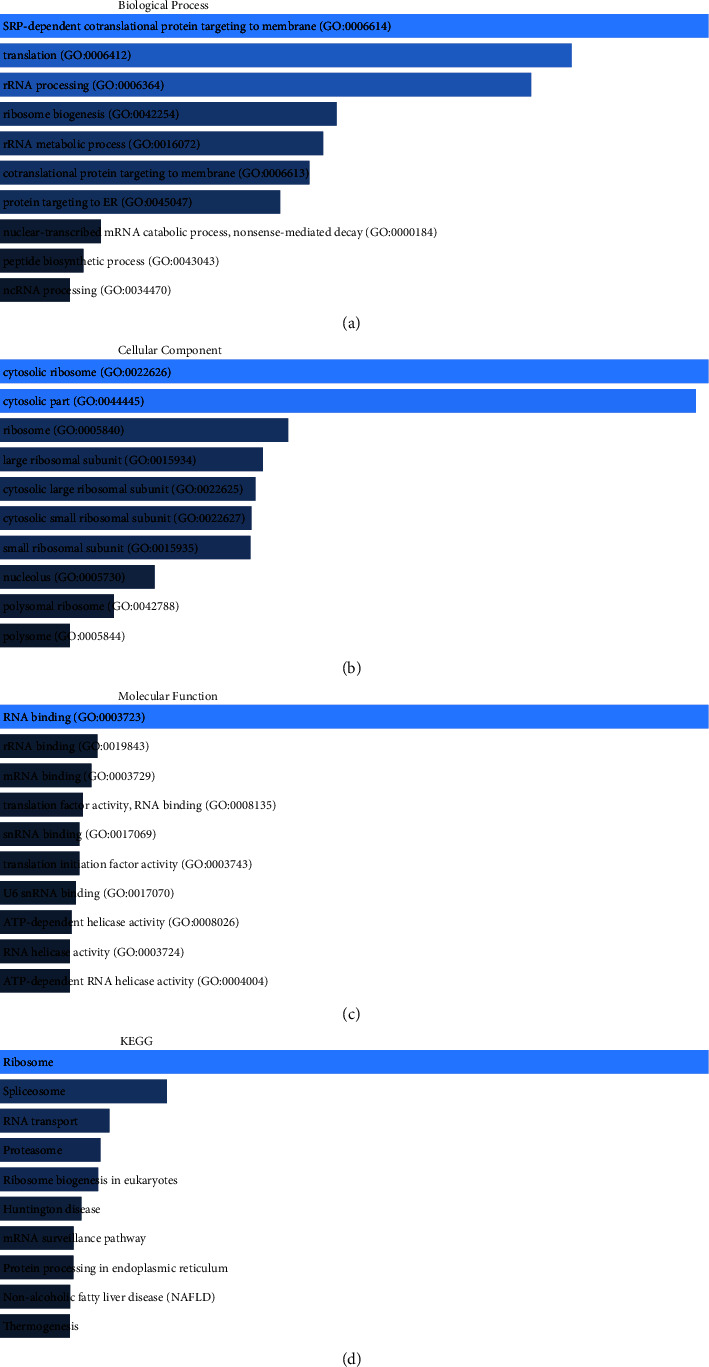
GO and KEGG analysis. (a–c) GO analysis of HM13-associated genes in BP, CC, and MF. (d) KEGG analysis of HM13-associated genes.

GO analysis showed that these genes could be classified into some pivotal BPs, including SRP-dependent cotranslational protein targeting to membrane, translation, cytosolic ribosome, cytosolic part, RNA binding, and rRNA binding (Figure 4(a)).  Figure 4(b) shows the GO analysis of the MFs related to these genes, comprising cytosolic ribosomes (including large ribosomal subunit), cytosolic part, and ribosome (including large ribosomal subunit).  Figure 4(c) shows the GO analysis of the CCs for these genes, comprising RNA (rRNA, mRNA) binding and translation factor activity. KEGG analysis revealed that these genes primarily participated in thermogenesis, nonalcoholic fatty liver disease, protein processing in the endoplasmic reticulum, the mRNA surveillance pathway, and Huntington's disease (Figure 4(d)).

### 3.5. Reduced HM13 Expression Inhibited LIHC Cell Proliferation, Migration, and Invasion

**Figure 5:**
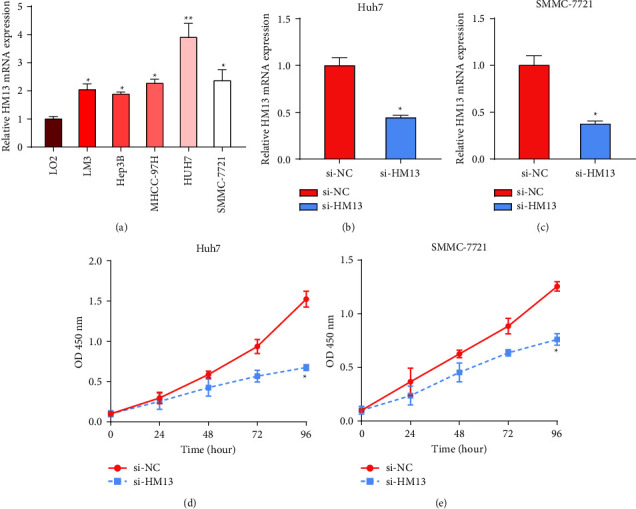
Assessment of reduced HM13 on LIHC cell proliferation. (a) qRT-PCR analysis of HM13 expression in LIHC cell lines and normal cells LO2 (^*∗*^*P* < 0.05; ^*∗∗*^*P* < 0.01). (b–c) To measure HM13 expression in HUh7 cells and SMMC-7721 cells by si-HM13 (^*∗*^*P* < 0.05). (d–e) To analyse the influence of reduced HM13 on cell proliferation of HUH7 and SMMC-7721 cells (^*∗*^*P* < 0.05).

**Figure 6:**
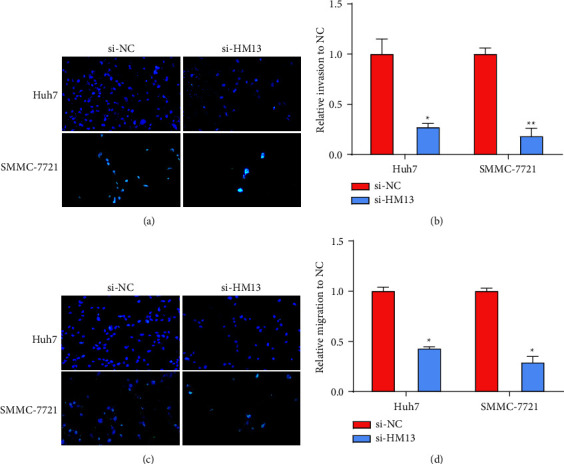
To evaluate the impacts of decreased HM13 on LIHC cell invasion and migration. (a–b) Evaluation of the influence of reduced HM13 on LIHC cell invasion (^*∗*^*P* < 0.05; ^*∗∗*^*P* < 0.01). (c–d) Validation of the effect of ablated HM13 on LIHC cell migration (^*∗*^*P* < 0.05).

We carried out CCK-8 and Transwell assays to explore HM13's function in LIHC. We chose Huh7 and SMMC-7721 cells in the following studies since they highly expressed HM13 compared with the other cell lines (Figure 5(a)). qRT‒PCR assays showed that HM13 was greatly reduced in Huh7 and SMMC-7721 cells after si-HM13 transfection (Figures 5(b) and 5(c)). The CCK-8 assay demonstrated that reducing HM13 resulted in a decrease in LIHC cell proliferation (Figures 5(d) and 5(e)). We also arrived at a similar conclusion that ablating HM13 hindered cell invasion (Figures 6(a) and 6(b)) and migration (Figures 6(c) and 6(d)) by the Transwell assay.

## 4. Discussion

LIHC and cholangiocarcinoma (CHOL) are common primary liver cancers worldwide [12, 13]. LIHC is a highly invasive and complex tumour disease caused by multiple aetiologies, consisting of altered cell behaviour of the neoplasm, the vascular system, and other causes [14, 15]. LIHC is one of the most common and deadly cancers in humans, so it is still a major challenge to global public health [16]. At present, the biological mechanism of liver cancer is unclear [17]. Nevertheless, Zhu et al. found that the interplay of chromosome 8 open reading frame 4 (C8orf4) with N2ICD could inhibit the Notch signalling pathway and negatively regulate liver cancer stem cell self-renewal [18]. Hu et al. found that linc00511 aggravated the LIHC process by accelerating cell proliferation and migration of neoplasms [19]. Here, we found that HM13 expression was obviously higher in LIHC than in normal tissues.

Li et al. confirmed that the protein phosphatase Mg2+/Mn2+-dependent 1D (PPM1D) was a biomarker for LIHC prognosis [20]. Chen et al. found that collagen triple helix repeat containing 1 (CTHRC1) was a probable biomarker for LIHC prognosis [21]. Jiao et al. showed that oxoglutarate dehydrogenase *L* (OGDHL) could be considered an indicator of LIHC diagnosis and prognosis [22]. To date, few studies have examined the association of HM13 expression with the prognosis of LIHC patients. To this end, we used a GEPIA dataset and a KM-plotter dataset to reveal the link between the expression of HM13 and poor prognosis in LIHC patients. The survival curve showed that highly expressed HM13 exhibited a significant association with poor prognosis in LIHC patients. These genes may be novel markers for predicting LIHC prognosis and new targets for immunotherapy, but further basic and clinical laboratory identification is needed.

HM13 is also named H13, SPP, IMP1, PSL3, IMPAS, SPPL1, PSENL3, IMPAS-1, and MSTP086, and it has widespread expression in human thyroid and salivary gland tissues [10]. Regarding the current studies on HM13, related disease studies are few. Further studies on HM13 expression and function in various diseases remain to be conducted. In this study, we explored the role of HM13 in LIHC through bioinformatics analysis and functional experiments. To further evaluate the integrity of our data, the GEPIA database was used to search for HM13-related genes. Our research screened 1,000 HM13-related genes. Bioinformatics analysis suggested that these genes were linked to rRNA processing ribosome biogenesis, spliceosome, Huntington's disease, and ATP-dependent helicase activity. The abovementioned pathways were reported to act as primary modulators of human cancer. For example, the results of the study offered genetic proof that the prostate carcinoma susceptibility gene ElaC ribonuclease *Z* 2 (ELAC2) perhaps participated in RNA processing, particularly rRNA processing and mitochondrial function [23]. The biosynthesis of ribosomes is necessary for cell growth and proliferation and is usually increased in carcinoma [24]. As Huang et al. demonstrated, Huntington's disease, a deadly neurodegenerative disease, was induced by amplification of the CAG repeats in the huntingtin genes [25]. Esmee Koedoot et al. demonstrated that obtaining a better understanding of the role of the spliceosome in the development of carcinoma could be conducive to developing strategies for treating carcinoma patients [26]. Li et al. showed that helicase, POLQ-like (HELQ) helicase could be applied to treat ovarian carcinoma [27]. Predicting protein analysis showed that HELQ mainly possessed ATP-dependent helicase activity and participated in DNA repair. These results showed that HM13 did have a crucial role in LIHC progression. The results of in vitro functional analysis showed that HM13 promoted LIHC cell proliferation, migration, and invasion. Our study showed that HM13 might act as an oncogene in LIHC and induce its proliferation, invasion, and migration.

Taken together, these results suggest that HM13 may be a promising candidate biomarker for LIHC diagnosis and prognosis. Reduction of HM13 resulted in inhibition of LIHC cell proliferation, migration, and invasion. Inhibition of HM13 expression may be an effective approach to improve the prognosis and treatment of LIHC. Overall, this study has certain significance for LIHC research and provides new ideas and insights for the diagnosis and treatment of LIHC. However, the study also has limitations. The sample size was relatively small, and the exact mechanism of HM13 in LIHC clinicopathological staging and prognosis remains to be explored. Further studies should focus on evaluating the relationship between serum HM13, efficacy, and prognosis by large-sample-size in vitro and in vivo studies.

## 5. Conclusion

Our current research first showed that HM13 is overexpressed in LIHC and that its reduction inhibits LIHC cell proliferation and metastasis. These results indicate that HM13 could be a biomarker for LIHC. Kaplan‒Meier plotter and GEPIA dataset analyses showed that high expression of HM13 was related to shorter OS, DFS, and DSS. Through GO and KEGG analyses of HM13-associated genes, we found that these genes greatly participated in rRNA processing, ribosome biogenesis, and other processes. The CCK-8 and Transwell assays showed that ablating HM13 resulted in reduced cell proliferation, migration, and invasion of LIHC. Our findings are beneficial for uncovering new targets for LIHC patient treatment.

## Data Availability

The datasets used and/or analysed during the current study are available from the corresponding author upon reasonable request.

## Abbreviation list

LIHC: Liver hepatocellular carcinoma
HM13: Histocompatibility minor 13
GEPIA: Gene Expression Profiling Interactive Analysis
OS: Overall survival
DFS: Disease-free survival
DSS: Disease-specific survival
GO: Gene ontology
KEGG: Kyoto encyclopedia of genes and genomes
CCK-8: Cell Counting kit-8
KM: Kaplan–Meier
BP: Biological process
CC: Cell components
MF: Molecular function
DAVID: Database for annotation, visualisation, and integrated discovery
ATCC: American Type Culture Collection
RPMI-1640: Roswell Park Memorial Institute 1640
FBS: Foetal bovine serum
DMEM: Dulbecco modified eagle medium
RT-PCR: Real-time polymerase chain reaction
SYBR: Synergy brands
GAPDH: Glyceraldehyde-3-phosphate dehydrogenase
DAPI: Diamidino-phenyl-indole
SPSS: Statistical Product and Service Solutions
ANOVA: Analysis of variance
AML: Acute myeloid leukaemia
Lung_AC: Lung adenocarcinoma
Lung_SC: Lung sarcomatoid carcinoma
Renal_CC: Renal clear cells
Renal_CH: Renal congenital hydronephrosis
Renal_PA: Renal pretubular aggregate
Uterus_CS: Uterus caesarean section
Uterus_EC: Uterus endometrial cancer
CHOL: Cholangiocarcinoma
C8orf4: Chromosome 8 open reading frame 4
PPM1D: Protein phosphatase, Mg2+/Mn2+ dependent 1D
CTHRC1: Collagen triple helix repeat containing 1
OGDHL: Oxoglutarate dehydrogenase L
ELAC2: ElaC ribonuclease *Z* 2
CAG: Coronary angiography.
HELQ: Helicase, POLQ-like.
